# The predictive performance of the uric acid-to-high-density lipoprotein cholesterol ratio (UHR) for cardiovascular diseases in cancer patients: A cross-sectional study from NHANES

**DOI:** 10.1097/MD.0000000000046732

**Published:** 2026-01-02

**Authors:** Yuning Gu, Chuanran Qin

**Affiliations:** a School of Pharmacy, Heilongjiang University of Chinese Medicine, Harbin, Heilongjiang Province, China.

**Keywords:** cancer, cardiovascular disease (CVD), NHANES, risk prediction, uric acid-to-high-density lipoprotein cholesterol ratio (UHR)

## Abstract

Cancer survivors are at an elevated risk of cardiovascular disease (CVD), imposing a significant burden on healthcare systems. The uric acid-to-high-density lipoprotein cholesterol ratio (UHR), a novel biomarker, has demonstrated predictive ability for cardiovascular events. However, whether UHR can effectively predict the incidence of CVD in cancer patients remains unclear. This study was based on National Health and Nutrition Examination Survey data (1999–2020), from which we constructed a nationally representative cross-sectional cohort of cancer patients. After rigorous screening, 4690 cancer patients were included in the final analysis. Weighted logistic regression models were employed to assess the association between UHR and CVD risk, with additional analyses using restricted cubic spline regression model to evaluate potential non-linear relationships. Subgroup analyses were conducted to explore interaction effects of various covariates on the relationship between UHR and CVD risk. Finally, Receiver Operating Characteristic (ROC) curve was applied to evaluate the predictive efficacy of UHR. The risk of CVD increased progressively with higher UHR levels. In fully adjusted models, each 1% increase in UHR was associated with a 3% higher risk of CVD (OR: 1.03, 95% CI: 1.01–1.05,  = =0.003). The restricted cubic spline regression model demonstrated a linear positive correlation between UHR and CVD risk. Subgroup analyses indicated that the impact of UHR on CVD risk was more pronounced in cancer patients who were former smokers or had no history of diabetes. Receiver Operating Characteristic (ROC) curve analysis showed that UHR had a modest discriminative ability in identifying CVD occurrence in cancer patients. This study offers novel evidence that UHR exhibited modest discriminative capacity and may function as a supplementary marker of CVD occurrence in cancer populations. The conduction of additional prospective cohort studies is indicated to further substantiate these findings.

## 1. Introduction

Cancer is a complex group of diseases characterized by uncontrolled cell growth and invasion of surrounding tissues, with the potential to metastasize to distant organs.^[1]^ In 2022, there were nearly 20 million new cases of cancer, while 9.7 million people died from cancer.^[2]^ The incidence of cancer has been on the rise, imposing a heavy burden on global healthcare systems.^[3]^ Although the survival of cancer patients has significantly improved with the development of healthcare, the adverse prognosis brought by cancer, such as cardiovascular disease (CVD), is still not optimistic. Research shows that the incidence rate of CVD among cancer survivors is as high as 23.1.^[4]^ Therefore, it is necessary to explore simple indicators that can effectively identify the comorbidity status of cancer and CVD.

Emerging evidence highlights a complex interplay between cancer and CVD, with shared pathophysiological mechanisms including chronic inflammation, oxidative stress, and metabolic dysfunction.^[5]^ Uric acid-to-high-density lipoprotein cholesterol ratio (UHR), a novel biomarker, is defined as the ratio of serum uric acid (UA) to high-density lipoprotein cholesterol (HDL-C). Uric acid acts as a potent pro-oxidant and HDL-C exerts cardio-protective effects.^[6]^ Previous epidemiological studies have shown that UHR has a good predictive ability for CVD related events. UHR was associated with CVD mortality in patients with diabetes.^[7]^ In addition, UHR was also associated with atrial fibrillation in patients with nonalcoholic fatty liver disease. UHR seems to be associated with arterial stiffness.^[8]^ However, it is still unclear whether UHR can effectively predict the incidence of CVD in cancer patients.

In summary, we utilized the National Health and Nutrition Examination Survey (NHANES) data and established a large-scale cross-sectional study aimed at exploring the association between UHR and CVD prevalence in the cancer population. We hope to provide new insights into predicting CVD in the cancer population. However, little is known about the role of UHR in predicting CVD among cancer patients, particularly using recent NHANES data. This gap underscores the need for the present analysis.

## 2. Methods

### 2.1. The study design and population

**Figure 1.**
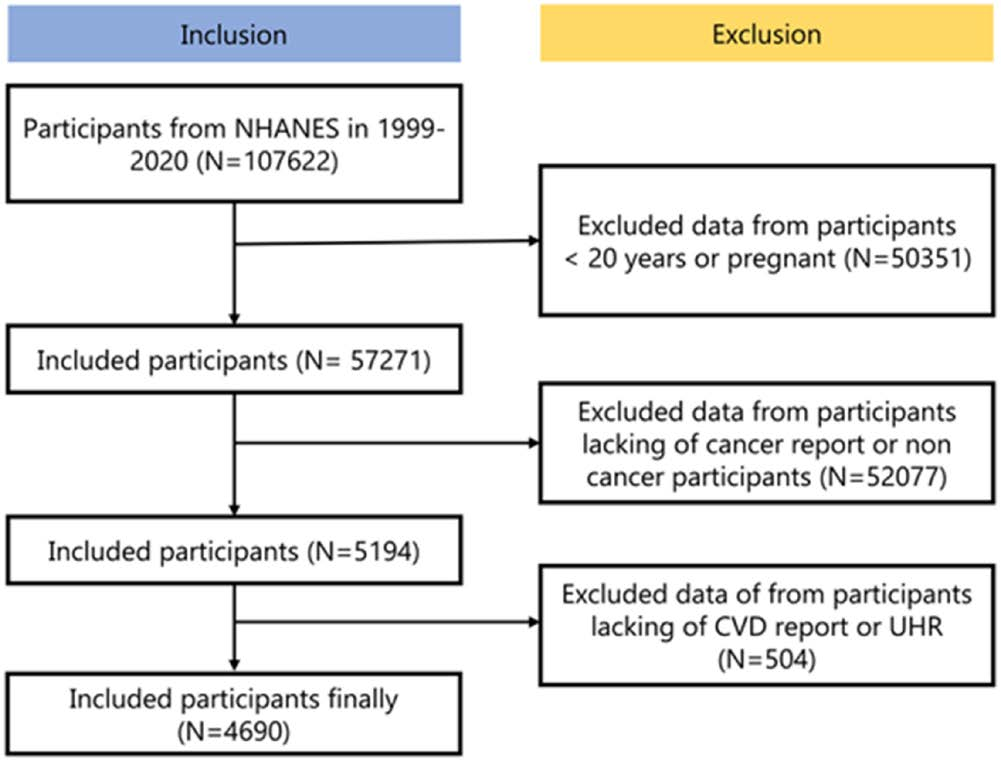
Enrollment pathway for participants. The diagram illustrates the sequential exclusion criteria applied to NHANES 1999–2020 data, beginning with 107,622 individuals. After removing participants under 20 years of age or pregnant (n = 50,351), those without valid cancer status (n = 52,077), and those missing CVD or UHR data (n = 504), a final analytical sample of 4690 cancer patients was included in the study. CVD = cardiovascular disease, UHR = uric acid-to-high-density lipoprotein cholesterol ratio.

NHANES is a countrywide survey that uses sophisticated multi-stage probability sampling methods to gather detailed data on the health and nutrition of the American people every 2 years.^[9]^ On a publicly accessible platform, the experimental design and associated NHANES data are available: www.cdc.gov/nchs/NHANES/. The data was publicly available and has been collected following ethical guidelines, including securing informed consent from all participants. The study population was initially drawn from the NHANES participants spanning 1999 to 2020, encompassing a total of 107,622 individuals. Through a rigorous screening process, we first excluded participants under 20 years of age or those who were pregnant (n = 50351), resulting in 57,271 eligible subjects. Subsequent exclusions were applied to individuals lacking complete cancer status reports or non-cancer participants (n = 52,077), followed by further exclusion of those without CVD records or unavailable UHR (n = 504). After these sequential exclusions, the final analytical population consisted of 4690 participants who met all inclusion criteria for the comprehensive analysis (Fig. 1).

### 2.2. Calculation of UHR

Detection of serum UA and HDL-C in the laboratory. For detailed testing methods, please refer to the official website of NHANES: www.cdc.gov/nchs/NHANES/. UHR (%) was calculated by dividing UA (mg/dL) by HDL (mg/dL) and multiplying by 100.^[10]^

### 2.3. Definition of cancer and cardiovascular disease

Cancer and CVD information both came from questionnaire surveys of research subjects. Interviewers with proper training hold direct dialogs with participants. Cancer status was determined by the NHANES interview item: “Has a doctor or other health professional ever told you that you had cancer or any kind of malignancy?” If they answered “Yes,” they were considered to have cancer.^[11]^ In addition, the researchers asked, “Have doctors or other health experts ever told you that you have congestive heart failure/coronary heart disease/angina pectoris/myocardial infarction/stroke? As long as participants answered “Yes” to one of these diseases, they were considered to have CVD.^[12]^ In NHANES, both cancer and CVD status were obtained through self-reported questionnaires. Although self-reported diagnoses may introduce some degree of misclassification, numerous studies using NHANES have demonstrated that this approach is widely accepted and provides reliable information for epidemiologic analyses. Prior validation studies have shown a generally good agreement between self-reported CVD and medical records, supporting its utility in large-scale population-based studies. Therefore, the use of questionnaire-based definitions of cancer and CVD in NHANES is considered a common and acceptable practice for epidemiologic research.^[13–15]^

### 2.4. Covariates

The covariates covered demographic characteristics, lifestyle factors, and clinical comorbidities, as detailed in Table S1, Supplemental Digital Content, https://links.lww.com/MD/R67.

### 2.5. Statistical analysis

All statistical analysis was conducted in R software (version 4.4.5) with two-tailed *P* value < .05 considered statistically significant. In this study, we utilized a mobile examination center MEC examination weight to stand for American population.^[16]^ To address the lack of covariates, aiming to optimize the sample size, we applied multiple imputation techniques in the “MICE” package.^[17]^ MICE is recommended for handling missing data in epidemiologic studies as it reduces bias and preserves statistical efficiency. In descriptive statistical analysis, we compared the differences in variable distribution between the subjects in 4 groups based on quartiles of UHR (Q1-Q4). For continuous variables, we reported weighted means with standard deviations (SD) and tested group differences using weighted *t* tests. Categorical variables were summarized as counts with weighted proportions and compared using weighted chi-square tests.^[18]^ First, we assessed the associations UHR and the risk of CVD in cancer patients by weighted logistic regression model. Odds ratio (OR) and 95% confidence interval (CI) were calculated in this section. UHR was used in continuous analyses and then quartiles of UHR were applied for categorical analyses with Q1 as a reference. We established three regression models: Model 1 (unadjusted), Model 2 (adjusted for demographics: age, sex, race, education, PIR and marital status), and Model 3 (adjusted for all covariates).^[19]^ To evaluate the potential nonlinear correlation between UHR and the risk of CVD, we established restricted cubic spline (RCS) regression models based on number of knots corresponding to the minimum Akaike information criterion.^[20]^ Subsequently, subgroup analyses were conducted to evaluate potential interaction effects between UHR and covariates on the risk of CVD.^[17]^ In the end, receiver operating characteristic (ROC) curve was applied to evaluate the predictive efficacy of UHR on the cancer individual.^[21]^ The larger the area under curve (AUC) value, the higher the predictive performance of UHR. Additionally, we conducted sensitivity analyses by further establishing unweighted logistic regression models. The results remained consistent with the primary analyses, supporting the robustness of our findings.

#### 2.5.1. Ethics statement

The NHANES protocol was approved by the National Center for Health Statistics Research Ethics Review Board (protocol numbers: 1999–2018). All participants provided written informed consent. Since the present study was a secondary analysis of publicly available de-identified data, additional ethical approval was not required.

## 3. Results

### 3.1. Characteristics of study objects

The basic characteristics of 4690 cancer participants from NHANES 1999 to 2020 were revealed in Table 1. Approximately half (42.5%) were male, and the largest proportion of participants were aged ≥ 60 years (62.5%). By comparing the differences in the distribution of different variables between on quartiles of UHR, we found that there were significant differences between the 4 groups in the variables except race and marital status. Importantly, the Q4 group had the significantly highest CVD incidence rate.

**Table 1:** Characteristics of cancer participants grouped by quartiles of UHR in NHANES 1999–2020.

Variables	Total	UHR, %				*P*
		Q1 (≤7.7)	Q2 (7.7–10.69)	Q3 (10.69–14.44)	Q4 (>14.44)	
n	4690	1174	1171	1181	1164	
Age, n (%)						<.001
<60 years	1283 (37.5)	402 (44.0)	311 (35.8)	314 (36.0)	256 (32.6)	
≥60 years	3407 (62.5)	772 (56.0)	860 (64.2)	867 (64.0)	908 (67.4)	
Gender, n (%)						<.001
Male	2242 (42.5)	261 (15.9)	501 (37.2)	666 (53.8)	814 (69.2)	
Female	2448 (57.5)	913 (84.1)	670 (62.8)	515 (46.2)	350 (30.8)	
Race, n (%)						.579
Mexican American	320 (2.3)	83 (2.4)	83 (2.5)	81 (2.2)	73 (2.2)	
Other Hispanic	234 (2.4)	59 (2.3)	63 (3.0)	58 (2.1)	54 (2.1)	
Non-Hispanic White	3289 (86.6)	834 (87.6)	809 (84.8)	822 (87.2)	824 (86.5)	
Non-Hispanic Black	630 (5.2)	139 (4.5)	161 (5.3)	165 (5.4)	165 (5.6)	
Other Race – Including Multi-Racial	217 (3.6)	59 (3.3)	55 (4.3)	55 (3.2)	48 (3.6)	
Education, n (%)						<.001
Less than high school	1030 (13.7)	212 (10.8)	254 (13.9)	256 (13.0)	308 (18.0)	
High school grad/GED or equivalent	1079 (22.8)	248 (21.1)	293 (24.2)	265 (22.8)	273 (23.6)	
Higher than high school	2581 (63.4)	714 (68.1)	624 (61.9)	660 (64.1)	583 (58.4)	
PIR, n (%)						<.001
≤1.3	1110 (15.9)	257 (13.4)	281 (17.6)	288 (17.2)	284 (16.0)	
1.3–3.5	1894 (36.3)	423 (33.0)	481 (37.7)	474 (35.1)	516 (40.4)	
>3.5	1686 (47.7)	494 (53.7)	409 (44.6)	419 (47.8)	364 (43.6)	
Marital status, n (%)						.104
Married/living with partner	2839 (65.3)	676 (63.1)	687 (63.8)	732 (66.2)	744 (68.8)	
Widowed/divorced/separated	1560 (28.8)	422 (31.2)	418 (30.4)	377 (28.4)	343 (24.3)	
Never married	291 (5.9)	76 (5.7)	66 (5.8)	72 (5.3)	77 (6.9)	
BMI, n (%)						<.001
<25 kg/m^2^	1334 (29.2)	596 (51.8)	343 (29.4)	235 (19.4)	160 (11.4)	
25-30 kg/m^2^	1669 (34.8)	389 (33.8)	441 (37.2)	424 (34.1)	415 (34.2)	
≥30 kg/m^2^	1687 (36.0)	189 (14.4)	387 (33.4)	522 (46.5)	589 (54.5)	
Smoking status, n (%)						<.001
Non smokers	2101 (46.1)	605 (53.3)	540 (45.9)	507 (43.5)	449 (39.9)	
Former smokers	1870 (37.9)	396 (32.0)	443 (37.1)	486 (39.1)	545 (44.9)	
Current smokers	719 (16.0)	173 (14.7)	188 (17.0)	188 (17.4)	170 (15.2)	
Drinking status, n (%)						<.001
Non drinkers	1991 (35.6)	422 (28.7)	511 (39.0)	481 (34.4)	577 (42.0)	
Current moderate drinkers	2474 (58.2)	654 (61.7)	615 (55.8)	653 (60.9)	552 (53.5)	
Current heavy drinkers	225 (6.2)	98 (9.5)	45 (5.2)	47 (4.7)	35 (4.5)	
Diabetes, n (%)						<.001
Yes	887 (15.4)	125 (7.1)	177 (12.5)	253 (18.6)	332 (25.7)	
No	3803 (84.6)	1049 (92.9)	994 (87.5)	928 (81.4)	832 (74.3)	
Hypertension, n (%)						
Yes	2615 (50.3)	525 (38.4)	607 (45.9)	691 (53.5)	792 (66.4)	<.001
No	2075 (49.7)	649 (61.6)	564 (54.1)	490 (46.5)	372 (33.6)	
TC, mg/dL, mean (SD)	197.15 (45.06)	206.91 (37.99)	198.81 (44.61)	193.34 (44.08)	187.27 (51.76)	<.001
CVD, n (%)						<.001
Yes	1176 (20.4)	201 (13.2)	259 (20.3)	285 (20.1)	431 (29.9)	
No	3514 (79.6)	973 (86.8)	912 (79.7)	896 (79.9)	733 (70.1)	

### 3.2. Association between UHR and CVD in cancer patients

Table 2 displayed the results of weighted logistic regression models. The risk of CVD increased gradually with a higher level of UHR in the three models. In Model 3, after taking all covariates in account, the risk of CVD increased by 3% with every percentage increase in UHR in continuous analyses (OR: 1.03, 95% CI: 1.01–1.05, *P* = .003). In categorical analyses, the objects Q4 group of UHR had a higher risk of CVD compared with Q1 group with the OR (95% CI) of 1.36 (1.02–1.81) and the linear trend was marginally obvious (*P* for trend = .053). The association was marginal (*P* = .053), suggesting a borderline statistical significance.

**Table 2:** The association of UHR with CVD in cancer patients in weighted logistic regression models.

	Model 1	*P*	Model 2	*P*	Model 3	*P*
	OR (95% CI)		OR (95% CI)		OR (95% CI)	
Continuous	1.07 (1.06, 1.09)	<.001	1.06 (1.04, 1.08)	<.001	1.03 (1.01, 1.05)	.003
Q1	Reference				Reference	
Q2	1.67 (1.30, 2.14)	<.001	1.4 (1.08, 1.81)	.013	1.19 (0.91, 1.54)	.201
Q3	1.65 (1.27, 2.13)	<.001	1.38 (1.05, 1.81)	.023	1.02 (0.75, 1.39)	.895
Q4	2.79 (2.21, 3.52)	<.001	2.19 (1.65, 2.90)	<.001	1.36 (1.02, 1.81)	.034
*P* for trend	<.001		<.001		.053	

Model 1 was unadjusted.

Model 2 was adjusted for age, sex, race, education, PIR, and marital status.

Model 3 was adjusted for age, sex, race, education, PIR, marital status, BMI, smoking status, drinking status, diabetes, hypertension, and TC level.

**Figure 2.**
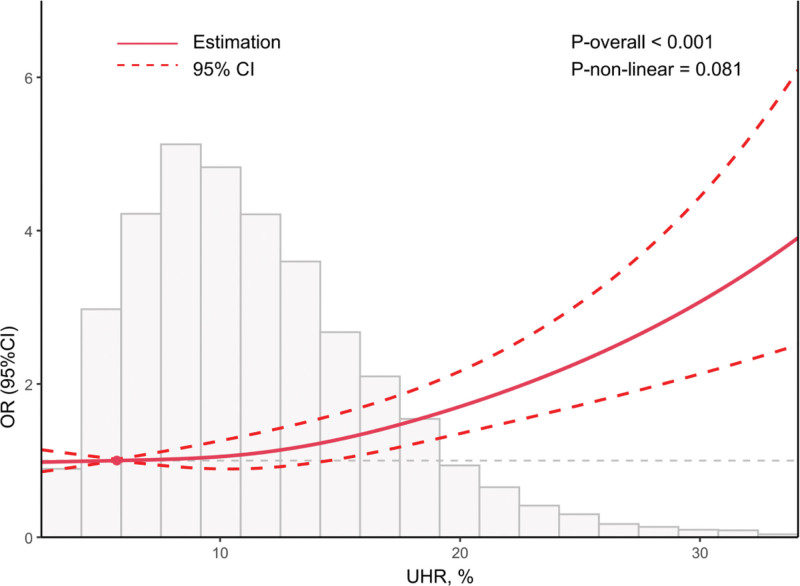
Association between UHR and CVD in cancer patients using RCS regression. The plot illustrates a linear positive relationship between UHR (%) and the OR of cardiovascular disease. The solid red line represents the estimated OR, while the dashed lines indicate the 95% confidence interval. The model was fully adjusted for age, sex, race/ethnicity, education, PIR, marital status, BMI, smoking status, drinking status, diabetes, hypertension, and total cholesterol. *P*-overall < 0.001; *P* for non-linearity = .081. BMI = body mass index, CI = confidence interval, CVD = cardiovascular disease, OR = odds ratio, PIR = poverty income ratio, RCS = restricted cubic spline, TC = total cholesterol, UHR = uric acid-to-high-density lipoprotein cholesterol ratio.

After that, we established RCS regression model with 3 knots to explore the non-linear relationship between UHR and CVD (Fig. 2). The RCS model demonstrated a linear positive correlation between UHR and the risk of CVD after considering all covariates (*P* for overall < .001, *P* for non-linear = .081).

CI = confidence interval, CVD = cardiovascular disease, OR = odds ratio, UHR = uric acid-to-high-density lipoprotein cholesterol ratio.

After that, we established RCS regression model with 3 knots to explore the non-linear relationship between UHR and CVD (Fig. 2). The RCS model demonstrated a linear positive correlation between UHR and the risk of CVD after considering all covariates (*P* for overall < .001, *P* for non-linear = .081).

### 3.3. Subgroup analyses

**Figure 3.**
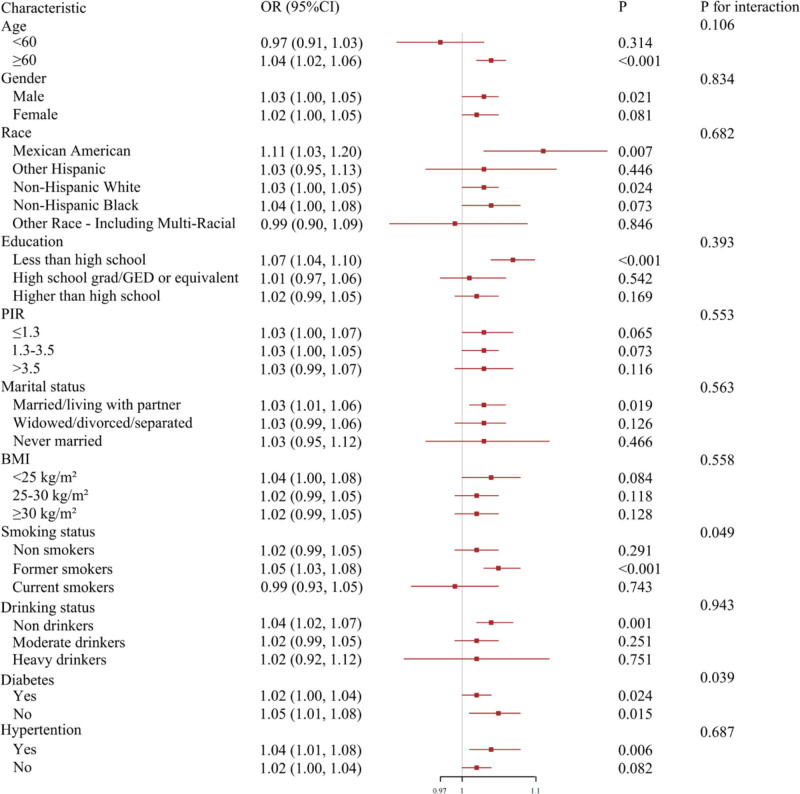
Subgroup analyses of the association between UHR and CVD in cancer patients. The forest plot presents ORs and 95% CIs for cardiovascular disease across multiple subgroups stratified by demographic and clinical characteristics. Interaction *P* values were calculated to assess effect modification. All subgroup models were fully adjusted for age, sex, race/ethnicity, education, PIR, marital status, BMI, smoking status, drinking status, diabetes, hypertension, and total cholesterol. BMI = body mass index, CI = confidence interval, CVD = cardiovascular disease, OR = odds ratio, PIR = poverty income ratio, TC = total cholesterol, UHR = uric acid-to-high-density lipoprotein cholesterol ratio.

Then, we explored the association between UHR and CVD in cancer patients according to different groups of covariates (Fig. 3). In the vast majority of groups, UHR was positively with the risk of CVD, in alignment with the aforementioned results. Subgroup analyses indicated significant interactions between UHR, smoking status, and diabetes history in relation to CVD risk (*P* for interaction < .05).

### 3.4. ROC curve of the predicted performance of UHR

**Figure 4.**
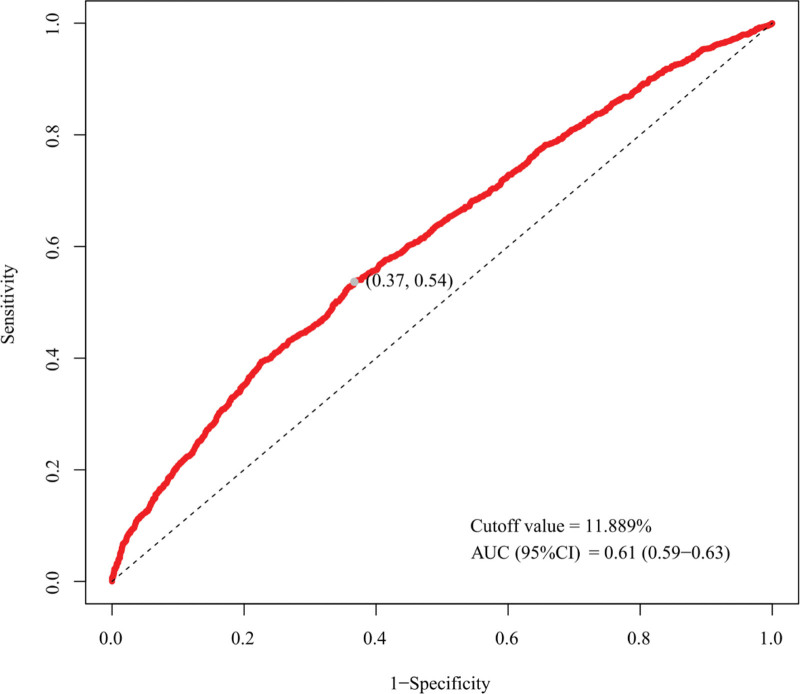
ROC curve for evaluating the discriminative ability of UHR for CVD among cancer patients. The AUC was 0.61 (95% CI: 0.59–0.63), with an optimal cutoff value of 11.889%. The corresponding sensitivity and specificity were 0.54 and 0.63, respectively, indicating only a modest discriminative capacity. AUC = area under the curve, CI = confidence interval, ROC = receiver operating characteristic.

Finally, the ROC curve was used to evaluate the predictive efficacy of UHR on CVD occurrence in patients with cancer (Fig. 4). UHR predicted the AUC (95% CI) of 0.61 (0.59, 0.63) and the cutoff value of UHR was 11.889%. The corresponding sensitivity and specificity were 0.54 and 0.63. The results indicated that UHR showed a modest discriminative ability in identifying CVD occurrence among cancer patients, with an AUC of 0.61, reflecting only a moderate level of classification performance.

### 3.5. Sensitivity analyses

The results of unweighted logistic regression models also indicated a positive correlation between UHR and CVD in cancer populations (Table 3).

**Table 3:** The association of UHR with CVD in cancer patients in logistic regression models.

	Model 1	*P*	Model 2	*P*	Model 3	*P*
	OR (95% CI)		OR (95% CI)		OR (95% CI)	
Continuous	1.08 (1.07, 1.09)	<.001	1.07 (1.05, 1.08)	<.001	1.04 (1.03, 1.06)	.005
Q1	Reference				Reference	
Q2	1.37 (1.12, 1.69)	.002	1.21 (0.98, 1.50)	.078	1.07 (0.86, 1.34)	.539
Q3	1.54 (1.26, 1.89)	<.001	1.33 (1.07, 1.65)	.009	1.05 (0.84, 1.32)	.675
Q4	2.85 (2.35, 3.46)	<.001	2.29 (1.86, 2.83)	<.001	1.59 (1.26, 2.00)	<.001
*P* for trend	<.001		<.001		<.001	

Model 1 was unadjusted.

Model 2 was adjusted for age, sex, race, education, PIR, and marital status.

Model 3 was adjusted for age, sex, race, education, PIR, marital status, BMI, smoking status, drinking status, diabetes, hypertension, and TC level.

BMI = body mass index, CI = confidence interval, CVD = cardiovascular disease, OR = odds ratio, PIR = poverty income ratio, RCS = restricted cubic spline, UHR = uric acid-to-high-density lipoprotein cholesterol ratio.

## 4. Discussion

To our knowledge, this is the first study on associations between UHR and incidence of CVD among cancer patients in American adults. We used the NHANES data and established a large-scale cross-sectional study. Weighted logistic regression model and RCS regression model indicated that higher UHR was associated with higher risk of CVD in cancer patients consistently. Subgroup analyses revealed that smoking status and diabetes might affect the relationship between UHR and CVD among cancer patients. ROC curve analysis further confirmed that UHR exhibited a modest discriminative ability (AUC = 0.61), suggesting that while UHR is statistically associated with CVD risk, its discriminative capacity as a standalone biomarker is limited. Sensitivity analyses further confirmed the robustness of the results. Therefore, UHR may serve as a supplementary marker for predicting CVD occurrence in cancer patients.

We have once again confirmed the close association between UHR and cardiovascular related outcomes. A retrospective cohort study showed a positive correlation between UHR and adverse cardiovascular outcomes in patients with chronic total coronary occlusion.^[22]^ A prospective cohort study in China has shown that higher UHR is associated with an increased risk of cardiac metabolic comorbidities in elderly Chinese individuals.^[23]^ Similarly, another large prospective cohort study found that accumulated UHR significantly increased the incidence of myocardial infarction events 10 years later.^[24]^ In addition, research from Japan has revealed that there is only a positive correlation between UHR and arterial stiffness when it is less than 14.25%, which was only significant in females.^[8]^ This provides clues for the threshold effect and gender specificity of UHR. Although UHR and cardiovascular events have been extensively explored, it is still unclear whether this association exists in cancer populations. We first explored the predictive value of UHR for CVD in cancer populations. Future prospective research and mechanism studies are needed to validate our conclusions.

Cancer and CVD are widely present due to shared mechanisms and complex interactions.^[25]^ Recent studies have revealed shared pathophysiological mechanisms between the two, including chronic inflammation, oxidative stress, and metabolic dysfunction. UA is a potent pro-oxidant that can promote inflammatory responses by increasing oxidative stress levels.^[26]^ Research has shown that higher serum uric acid levels are significantly correlated with elevated levels of reactive oxygen metabolite derivatives (d-ROM).^[27]^ High uric acid levels are associated with an increased risk of various cardiovascular diseases, including coronary heart disease, heart failure, and stroke.^[28,29]^ In addition, both experimental and population studies have consistently shown an association between UA and the onset of various cancers.^[30,31]^ High uric acid level is also closely related to metabolic diseases such as metabolic syndrome, diabetes and nonalcoholic fatty liver disease, which further increases the risk of CVD.^[32,33]^ In contrast, HDL-C has antioxidant and anti-inflammatory effects, which can protect the cardiovascular system and inhibit the progression of cancer.^[34–36]^ In addition, aging seems to be a common mechanism affecting cancer and CVD.^[37–41]^ HDL may antagonize atherosclerosis by regulating anti-aging proteins such as α–klotho.^[10]^ However, UA seems to increase the process of human aging, as confirmed by a study that serum UA significantly increases the biological age of elderly people.^[39]^ Therefore, UHR, as a comprehensive indicator, can reflect the balance between oxidative stress and inflammatory status in the body. A higher UHR value may indicate higher levels of oxidative stress in the body, while weaker anti-inflammatory and antioxidant capabilities increase the risk of cardiovascular disease.

In addition, subgroup analysis also found that smoking status and diabetes history may affect the relationship between UHR and cardiovascular disease. Specifically, for former smokers or cancer patients without a history of diabetes, UHR has a more significant impact on cardiovascular disease. This may be related to the regulatory effect of smoking and diabetes on oxidative stress and inflammatory reaction. Smoking has been proved to increase the level of oxidative stress, and diabetes is closely related to chronic inflammation.^[42]^ Therefore, in these specific populations, UHR may better reflect potential cardiovascular risks. Previous studies have proposed several inflammatory or metabolic markers, such as triglyceride-to-HDL ratio, neutrophil-to-lymphocyte ratio, systemic immune-inflammation index, and systemic inflammation response index, as predictors of cardiovascular risk. While these markers have shown utility in different populations, they often require multiple hematological parameters and relatively complex calculations. Although these markers are also associated with CVD, UHR is easier to obtain, requiring only two routine tests. By contrast, the UHR can be readily obtained from two routine biochemical tests (serum uric acid and HDL cholesterol), which are inexpensive and widely available. This simplicity makes UHR particularly attractive for clinical screening, especially in resource-limited settings, although its predictive value remains modest (AUC = 0.61 in our study).

This study has several strengths that enhance the validity and reliability of the findings. Firstly, this is the first study to explore the association between UHR and CVD in cancer patients, providing valuable clues for the occurrence of CVD in cancer patients. Secondly, the utilization of the NHANES data provides a large-scale, nationally representative sample, which allows for generalizability of the results to the broader population of American adults. Thirdly, the study employed some rigorous statistical approaches, including weighted logistic regression models, RCS model, subgroup analysis, and ROC curve to comprehensively explore the correlation between UHR and CVD and the predictive value of UHR in CVD in cancer patients.

Despite these strengths, several limitations should be acknowledged. Firstly, the cross-sectional design of NHANES precludes causal inference between UHR and CVD. Future prospective cohort studies are warranted to clarify the temporal and causal relationships. Secondly, the diagnosis of CVD relied on self-reported questionnaires, which may introduce misclassification bias. Although NHANES uses standardized interviews by trained personnel, recall error and underreporting cannot be fully excluded. Thirdly, although we adjusted for numerous covariates and performed sensitivity analyses with additional metabolic indicators (e.g., triglycerides, waist circumference), residual confounding remains possible. Furthermore, our study did not stratify analyses by cancer type or treatment modality. Since different cancers and therapies (e.g., chemotherapy, radiotherapy, and targeted therapy) may affect cardiovascular risk differently, this lack of stratification could obscure heterogeneity. Lifestyle and pharmacological factors (e.g., diet, urate-lowering drugs, statins, physical activity) were also not captured in NHANES and may contribute to unmeasured confounding. Finally, as the study population was limited to U.S. adults, external validation in diverse ethnic and geographic populations is needed to strengthen generalizability.

## 5. Conclusion

In summary, our study demonstrated a positive correlation between UHR and the risk of CVD in cancer patients among American adults. UHR showed modest discriminative capacity of CVD in cancer patients. Prospective and experimental studies are needed to validate this conclusion. These findings highlight the potential utility of UHR in risk stratification, although further validation in longitudinal studies is needed before clinical implementation.

## Acknowledgments

The authors thank the staff and participants of the National Health and Nutrition Examination Survey (NHANES) for generously sharing their time and data, which made this analysis possible.

## Author contributions

**Conceptualization:** Yuning Gu.

**Data curation:** Yuning Gu, Chuanran Qin.

**Formal analysis:** Yuning Gu.

**Funding acquisition:** Yuning Gu, Chuanran Qin.

**Investigation:** Yuning Gu.

**Methodology:** Yuning Gu.

**Project administration:** Yuning Gu, Chuanran Qin.

**Resources:** Yuning Gu.

**Software:** Yuning Gu, Chuanran Qin.

**Supervision:** Yuning Gu, Chuanran Qin.

**Validation:** Yuning Gu, Chuanran Qin.

**Visualization:** Yuning Gu, Chuanran Qin.

**Writing – original draft:** Yuning Gu, Chuanran Qin.

**Writing – review & editing:** Yuning Gu, Chuanran Qin

## Supplementary Material


