# Supplementary material for: The predictive performance of the uric acid-to-high-density lipoprotein cholesterol ratio (UHR) for cardiovascular diseases in cancer patients: A cross-sectional study from NHANES
Source: Medicine (Baltimore). 2026 Jan 2;105(1):e46732. doi: 10.1097/MD.0000000000046732 (PMC12778100; doi:10.1097/MD.0000000000046732)
Supplement: Supplementary file 1 [file medi-105-e46732-s001.pdf]

Supplement Table 1. Classification and definition of covariates.

Covariate Category	Covariate Name	Categories or source
Sociodemographic Variables	Gender	Male, Female
	Age	20–39 years, 40–59 years, ≥60 years
	Race	Mexican American, Other Hispanic, Non-Hispanic White, Non-Hispanic Black, Other Race (including multi-racial)
	Educational Level	Less than high school, High school graduate/GED or equivalent, Higher than high school
	Household Poverty-to-Income Ratio (PIR)	≤1.3, 1.3–3.5, >3.5
	Marital Status	Married/Living with a partner, Widowed/Divorced/Separated, Never married
Life Behavior Variables	Body Mass Index (BMI)	<25 kg/m², 25-30 kg/m², ≥30 kg/m²
	Smoking Status	Nonsmokers (<100 cigarette in lifetime), Former smokers (>100 cigarette in lifetime but no cigarette in current), Current smokers (>100 cigarette in lifetime and cigarette in current)
	Drinking Status	Nondrinkers (no alcohol), Moderate drinkers (≤2 drinks/day for male, ≤1 drink/day for female), Heavy drinkers (>2 drinks/day for male, >1 drink/day for female)
	Diabetes and hypertension	From the medical history obtained in survey questionnaire inquiries
	Total cholesterol (TC)	From the laboratory
